# The effect of parental diabetes prevention program participation on weight loss in dependent children: a prospective cohort study

**DOI:** 10.1186/s40842-023-00154-5

**Published:** 2023-12-10

**Authors:** Namratha Atluri, Joshua Thariath, Laura N. McEwen, Wen Ye, MinKyoung Song, William H. Herman

**Affiliations:** 1 University of Michigan Medical School, Ann Arbor, MI USA; 2 Department of Internal Medicine, University of Michigan, 1000 Wall Street, Room 6108, Ann Arbor, MI 48105 USA; 3 University of Michigan School of Public Health, Ann Arbor, MI USA; 4 School of Nursing, Oregon Health and Science University, Portland, OR USA

**Keywords:** National diabetes prevention program, Prediabetes, Children, Obesity

## Abstract

**Introduction:**

Obesity has reached epidemic proportions in children and adolescents in the United States. Children’s behaviors are strongly influenced by parental behaviors, and weight loss in parents is positively associated with weight changes in their overweight/obese children. Research is limited on how parents’ National Diabetes Prevention Program (DPP) participation affects the health outcomes of their dependent children. Analyzing the impact of parental DPP participation on weight loss in their dependent children may provide valuable insight into an important secondary benefit of DPP participation.

**Methods:**

In this study, we identified 128 adults with prediabetes who were offered the opportunity to participate in a DPP (*n* = 54 DPP participants and *n* = 74 DPP non-participants) and who had at least one child 3 to 17 years of age living with them. Age and BMI percentile for dependent children were collected from insurance claims data for 203 children (*n* = 90 children of DPP participants and *n* = 113 children of DPP non-participants). Parental practices related to diet and physical activity were assessed by surveys.

**Results:**

There were no significant changes in BMI percentiles of overweight or obese children (i.e. BMI percentile ≥ 50%) of DPP participants vs DPP non-participants with prediabetes over one-year. Parents who enrolled and did not enroll in the DPP did not report differences in their parenting practices related to diet and physical activity.

**Discussion:**

These results are not consistent with the literature that suggests parent-based interventions may influence their children’s weight trajectories. Limitations include small sample size, short time span of intervention, and limited availability of additional health/biographic data on dependent children. Future studies should collect primary outcome data on children, investigate whether there is a minimum duration of parental involvement and level of parental adherence, and assess the effect of parent–child dynamics on child weight trajectories.

## Introduction

While obesity has long been recognized as a major public health challenge, the global obesity epidemic only continues to grow. Childhood obesity is reaching alarming proportions in the US, with 18% of children and adolescents 2–19 years of age being obese [1–3]. In addition to being a major risk factor for hypertension, cardiovascular disease, joint disorders, gallbladder diseases, and depression, obesity is intricately linked to the rising prevalence of type 2 diabetes (T2D) in both children and adults [4, 5]. While T2D only accounted for 1–2% of prevalent diabetes in children in the 1990s (the majority being type 1 diabetes), now nearly 45% of diabetes in children is attributable to T2D [6]. These trends emphasize the need to focus on obesity prevention and treatment in children and adolescents.

Achieving weight loss in children is complex and is influenced by many factors including parental behaviors and the home environment [7, 8]. Prior studies have found that in the treatment of childhood overweight and obesity, parent-only weight loss interventions are either more effective or equally effective as child-only or parent–child interventions [9]. There is also evidence to suggest positive associations between parental health habits, such as conscious eating and vigorous physical activity, and their children’s health habits, demonstrating the value of parental modeling [7, 8, 10]. Family-centered weight interventions where parents and children both receive diet modification and physical activity reinforcement have been shown to be more effective than when only children receive the intervention [11, 12]. A systematic review of fourteen studies by Tomayko et al., and work by Boutelle et al. and Golan et al. showed that interventions targeting only parents might be as effective as or non-inferior to those targeting parents and children together in promoting child weight loss [11, 13, 14].

Several strategies have been proposed to address the epidemic of diabetes in the US including the National Diabetes Prevention Program (DPP). While they may vary in structure and delivery mode, DPPs provide intensive lifestyle interventions through public and private institutions focused on reducing the risk of developing diabetes [15, 16]. Recognizing research showing a 16% reduction in the risk of type 2 diabetes with each kilogram of body weight lost, the DPP’s main goal is weight loss. DPP participation has been shown to lead to approximately 5% weight loss for participants with prediabetes, thereby reducing the risk of diabetes and of downstream diabetes-related complications. However, research is limited on how DPP participation may affect the health outcomes of participants’ dependent children. Analyzing the effectiveness of parental DPP participation in facilitating weight loss in their children may provide valuable insight into the ability of DPPs to indirectly address childhood obesity and highlight important secondary benefits of DPP participation.

## Methods

Beginning in August 2015, a large research university in the Midwestern United States offered its self-insured employees, dependents, and retirees with prediabetes and obesity the option of participating in a one-year DPP at no out-of-pocket cost. The university used a tiered intervention strategy to identify individuals with prediabetes [17]. Between 2015 and 2018, individuals with prediabetes were sent letters encouraging them to join a DPP [17]. For this current study, baseline and one-year follow-up surveys were administered to all individuals with prediabetes who enrolled in a DPP and a random sample of those who had not enrolled.

**Fig. 1:**
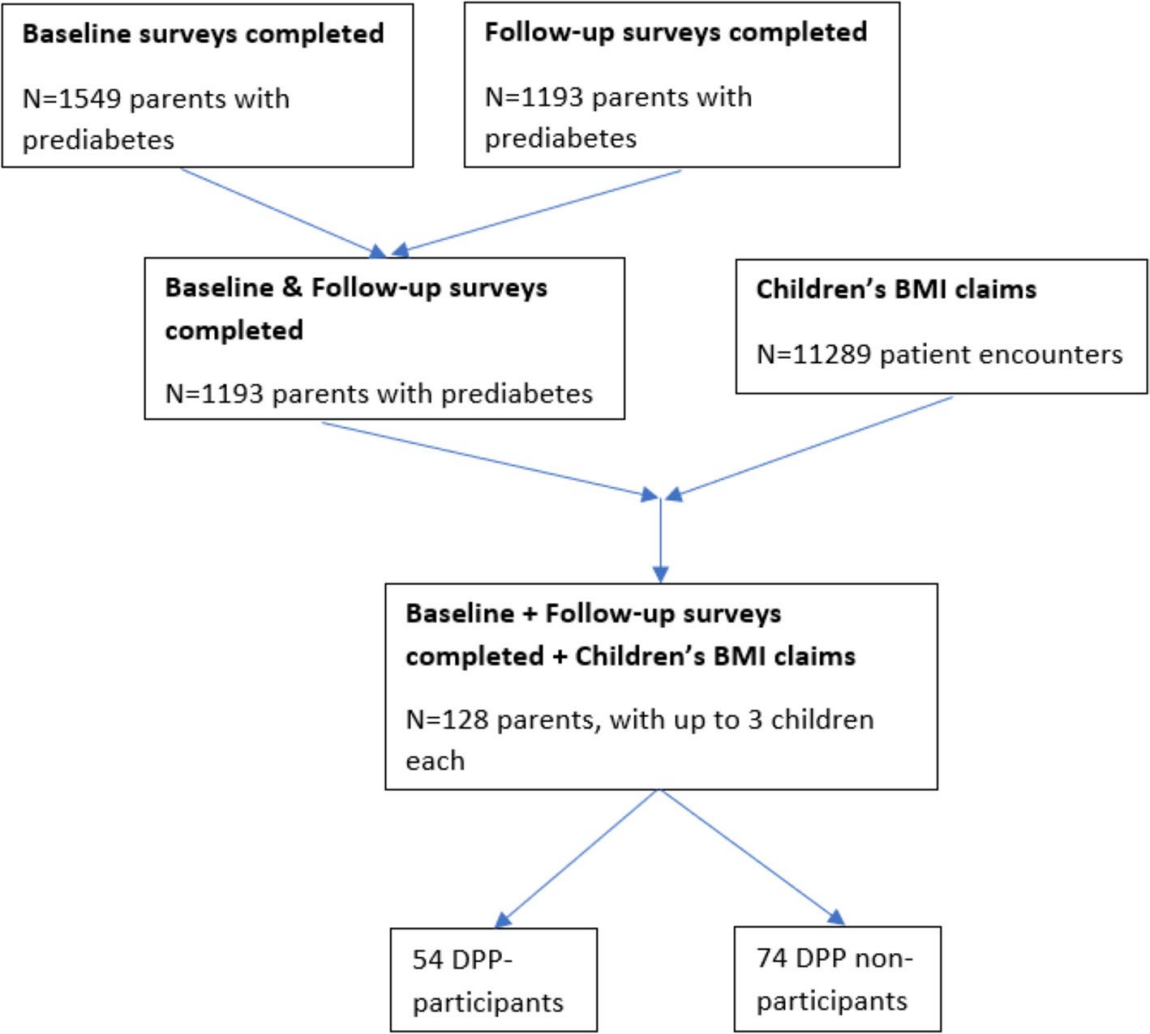
Consort diagram

Of the 1,193 survey respondents with prediabetes and both baseline and follow-up survey data, 128 individuals (*n* = 54 DPP participants and *n* = 74 DPP non-participants) reported having at least one child 3 to 17 years of age living with them (Fig. 1). Our primary study outcome was change in BMI percentile over one year in dependent children of parents with prediabetes who participated and did not participate in the DPP. Age and BMI percentile for dependent children were collected from insurance claims data for 203 children (*n* = 90 children of DPP participants and *n* = 113 children of DPP non-participants). Secondary outcomes of interest were parental survey data, which included questions about parenting practices related to diet and physical activity (See Fig. 3 in Appendix). For diet and physical activity, summed score maximums were 3 and 12 respectively, and mean score maximums were 1 and 4 respectively. To calculate the mean score, the sum score was divided by the number of questions. The higher the summed or mean score, the more often parents reported facilitating healthy diet and physical activity behaviors in their children.

## Data analysis

The dependent children were stratified according to their parents’ participation in the DPP. Each child’s baseline and follow-up BMI percentile, as well as ages when the BMI percentiles were collected, were compared between dependent children of DPP participants and non-participants with prediabetes to determine if there were any differences. Analyses were performed with SAS version 9.4 (SAS Institute, Cary, NC).

A linear mixed-effects regression model was used to characterize differences in BMI percentile trajectory between children of DPP participants and non-participants. All analyses used BMI percentiles taken ≤ 3 years after t = 0, defined as when parents with prediabetes were mailed letters notifying them of their eligibility to participate in the DPP, plus the median number of days before enrollment in the DPP program (for participants). We focused on children with BMI percentiles ≥ 50 because a BMI percentile of 50 is correlated with adiposity measures and weight loss might be appropriate for these children [18].

The model used a one-knot linear spline to test the change of BMI percentile after t = 0. A random intercept and random slope were included in the model to account for within-subject correlation.

$$\mathrm{BMI\;\% }=\mathrm{ Intercept }+\mathrm{ a}*\mathrm{Month }+\mathrm{ b}*\mathrm{Month\;}(\mathrm{Plus\; function})$$

The coefficient associated with the Month variable can be interpreted as the change rate of BMI before t = 0. The coefficient associated with the Month (Plus function) variable can be interpreted as the difference between the change rate of BMI percentile after and before t = 0.

In addition, we studied the effects of parental sex, parental percent weight loss, and parental DPP attendance on change in children’s BMI percentile. We added parent’s sex, percent weight loss, DPP attendance, and their interactions with two time variables to the linear mixed effects model. We conducted the analysis stratified by average BMI percentile < 50% or ≥ 50% before t = 0.

## Results

The characteristics of the 203 dependent children are shown in Table 1. There is no statistically significant difference between the children’s ages when the baseline or follow-up BMI percentile were collected for children of DPP participants and non-participants nor in their actual BMI percentiles. However, there were slight differences between DPP participants' children and non-participants' children. Thirty-four percent of DPP participants' children had baseline BMI percentiles above the 85th percentile, while only 27% of non-participants’ children had baseline BMI percentiles above the 85th percentile. Twenty-eight percent of participants’ children had baseline BMI percentiles below the 50th percentile, while 35% of non-participants’ children had baseline BMI percentiles below the 50th percentile.

**Table 1:** BMI Percentiles of children of DPP participants and non-participants

	Total (*n* = 203)	Children of DPP-participants (*n* = 90)	Children of DPP-non-participants (*n* = 113)
Order of dependent children with data			
First child	127	54	73
Second child	61	29	32
Third child	15	7	8
Mean age of child at first BMI percentile assessment (years)	10.8 ± 4.4	10.7 ± 4.2	10.9 ± 4.6
Mean age of child at last BMI percentile assessment (years)	12.8 ± 4.0	12.9 ± 3.7	12.6 ± 4.3
Distribution of BMI percentile at first assessment			
≥ 85	62(31%)	(31%)	(31%)
≥ 65 and < 85	(24%)	(20%)	(29%)
≥ 50 and < 65	(14%)	(14%)	(14%)
< 50	(32%)	(25%)	(39%)
Mean BMI percentile at first assessment	62.8 ± 29.3	65.0 ± 28.7	61.1 ± 29.8
Mean BMI percentile at last assessment	65.4 ± 28.8	69.5 ± 27.6	62.2 ± 29.5

For children of DPP participants, BMI percentile increased by 0.05% per month before t = 0 (*p*-value not significant). After t = 0, BMI percentile increased by 0.03% per month (*p*-value not significant).

For children of DPP non-participants, BMI percentile increased by 0.02% per month (*p*-value not significant). After t = 0, BMI percentile decreased by 0.12% (*p*-value not significant).

**Fig. 2:**
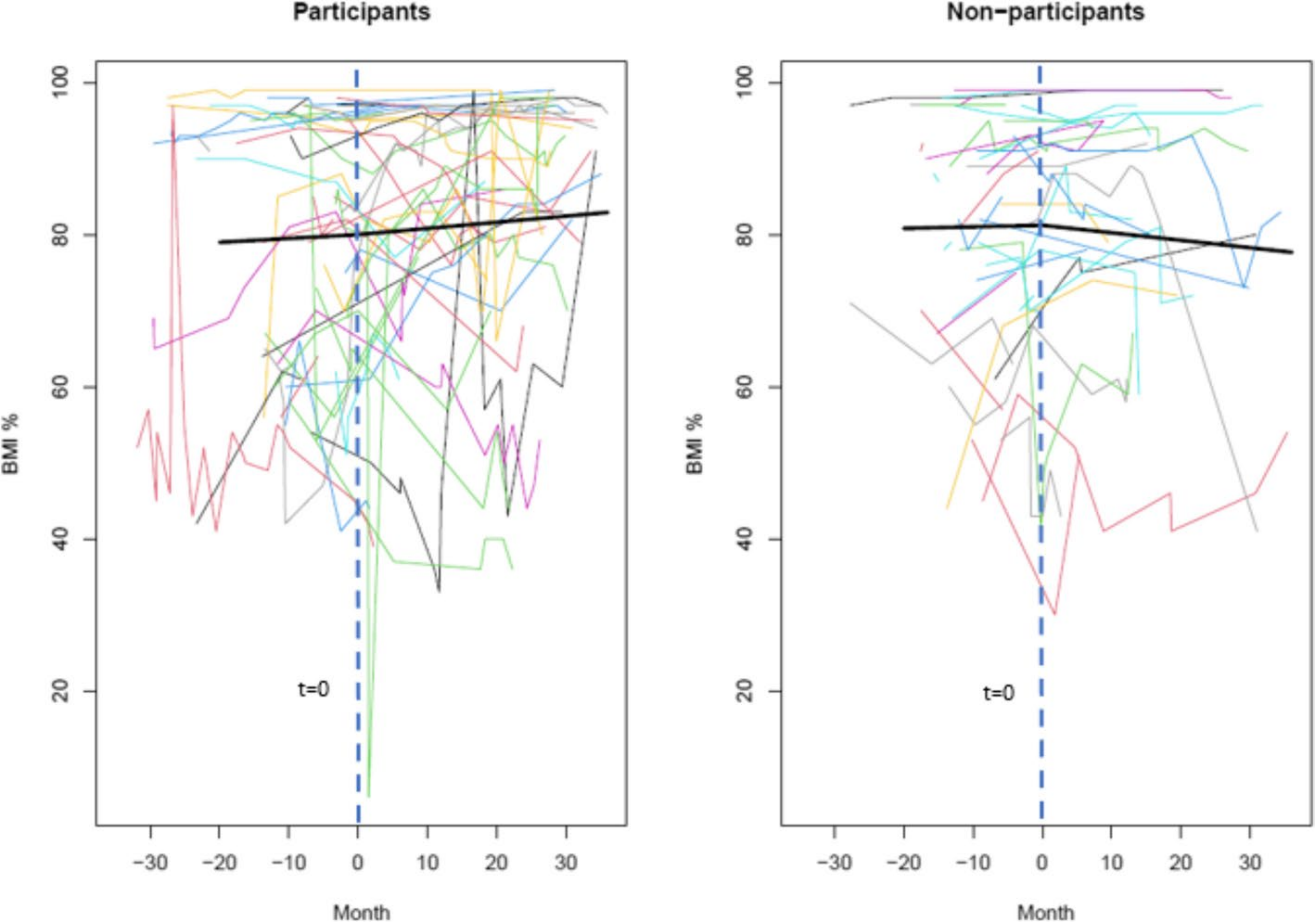
BMI Percentile Trajectories Before and After t = 0 (denoted as month 0) for Dependent Children with Baseline BMI ≥ 50th percentile

**Fig. 3:**
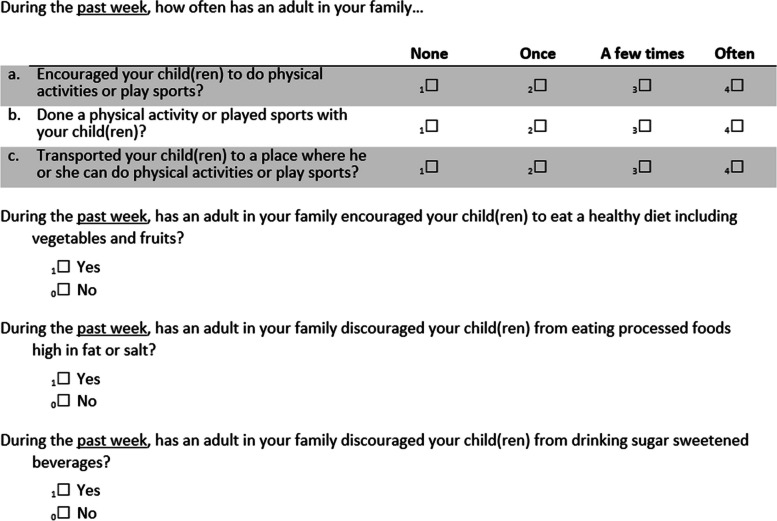
Survey questions assessing parenting practices related to physical activity and healthy diet

Figure 2 shows the change of BMI percentile over time before and after t = 0 for children of DPP participants and non-participants. The bolded lines show the overall trend for each group and each colored line shows an individual child’s data.

Parental attributes including sex, weight loss during the DPP (greater or less than or equal to 5%), and attendance of DPP sessions (fewer than 22 sessions or ≥ 22 sessions) were not associated with change in children’s weight over time.

Information about parenting practices related to diet and physical activity were reported by 128 parents. Although there was a trend, there was no significant difference in reported parenting practices related to diet between DPP participants (sum diet score 2.7) and non-participants (sum diet score 2.4). There was also no significant difference in reported parenting practices related to physical activity between DPP participants (sum physical activity score 8.6) and non-participants (sum physical activity score 9.0).

## Discussion

Parental participation in the DPP did not affect the BMI percentiles of their dependent children with BMI percentile ≥ 50th percentile at baseline. At baseline, there was no significant difference in the average BMI percentile of dependent children of DPP participants with prediabetes compared to those of dependent children of DPP non-participants with prediabetes. At follow-up, no significant change in BMI percentile was noted in either group of children. There were also no significant differences in parenting practices related to diet and physical activity between DPP participants and non-participants (Table 2).

**Table 2:** Diet and physical activity survey scores reported by DPP participants and DPP non–participants

	Total *N* = 128	Participant *N* = 54	Non-participant *N* = 74	***p*-value**
Unweighted sum diet score	2.5 ± 0.8	2.7 ± 0.7	2.4 ± 0.8	0.0529
Mean diet score	0.84 ± 0.26	0.90 ± 0.22	0.80 ± 0.28	0.0529
Unweighted sum physical activity score	8.8 ± 2.5	8.6 ± 2.6	9.0 ± 2.3	0.3344
Mean physical activity score	2.9 ± 0.8	2.9 ± 0.9	3.0 ± 0.8	0.3344

These results are not consistent with the literature that suggests that parent-based interventions may influence children’s weight loss. Prior work has shown parental encouragement and modeling of healthy behaviors is vital to reducing child and adolescent obesity risk [7, 8]. There is also evidence that joint parent–child interventions and parent-only interventions lead to direct effects on children’s weight loss [8, 10–14]. Given these past studies and the demonstrated efficacy of DPPs in promoting the adoption of healthy diet and activity habits [17], parental participation in the DPP might lead to improvements in BMI percentile of their dependent children. However, in this study, parental participation in the DPP was not specifically designed to influence children’s BMI percentile. Rather, this study sought to determine collateral benefits to children of DPP participants, simply by living in the same household.

While the results do not support this hypothesis, there are several limitations to this study that may have contributed to the negative results. The small sample size, short time span of the intervention, and limited availability of health/biographic data on dependent children limited our ability to assess the effect of parental DPP participation on dependent children’s weight trajectories. While successful parent or family-based interventions were able to better characterize parent–child relationship dynamics and other possible confounding factors, this study was not able to due to unavailability or incompleteness of such data. In addition, the provider-reported BMI percentiles used for this analysis, which were ascertained from health maintenance claims data, may not always be accurate. Therefore it is difficult to quantify what, if any, effect parent–child factors had on this study’s results. Future studies should focus on collecting primary data on children, investigating the minimum duration of parental interventions before effects on children can be detected, how the level of parents’ adherence to and success with programs such as DPPs could influence their children’s weight trajectories, and how parent–child dynamics impact the ability of parent-based interventions in promoting children’s weight loss.

## Conclusion

There is an urgent need to address the exponentially growing childhood obesity epidemic in the United States. Given the influence parental behaviors can have on shaping children’s behaviors, this study sought to explore the effect a weight loss intervention (DPP) focused on parents can have on their dependent children. While this study found no significant change over one year in BMI percentiles of dependent children whose parents participated in a DPP, it does not mean parent-based interventions have no utility in affecting child weight loss. Rather, the results of this study suggest that further research should focus on collecting more primary data on children’s health outcomes beyond simply BMI percentiles and understanding the complex parent–child dynamics that impact weight loss behaviors.

## Data Availability

Not applicable.

## Abbreviations

DPP: Diabetes Prevention Program
T2D: Type 2 Diabetes
BMI: Body Mass Index
